# Role of radiation oncology in modern multidisciplinary cancer treatment

**DOI:** 10.1002/1878-0261.12712

**Published:** 2020-06-22

**Authors:** Vincenzo Valentini, Luca Boldrini, Silvia Mariani, Mariangela Massaccesi

**Affiliations:** ^1^ Dipartimento di Diagnostica per Immagini, Radioterapia Oncologica ed Ematologia UOC Radioterapia Oncologica Fondazione Policlinico Universitario “A. Gemelli” IRCCS Rome Italy; ^2^ Istituto di Radiologia Fondazione Policlinico Universitario A. Gemelli IRCCS‐Università Cattolica Del Sacro Cuore Rome Italy

**Keywords:** modern radiotherapy, multidisciplinary cancer treatment

## Abstract

Cancer care is moving from a disease‐focused management toward a patient‐centered tailored approach. Multidisciplinary management that aims to define individual, optimal treatment strategies through shared decision making between healthcare professionals and patient is a fundamental aspect of high‐quality cancer care and often includes radiation oncology. Advances in technology and radiobiological research allow to deliver ever more tailored radiation treatments in an ever easier and faster way, thus improving the efficacy, safety, and accessibility of radiation therapy. While these changes are improving quality of cancer care, they are also enormously increasing complexity of decision making, thus challenging the ability to deliver quality affordable cancer care. In this review, we provide an updated outline of the role of radiation oncology in the modern multidisciplinary treatment of cancer. Particularly, we focus on the way some developments in key areas of cancer management are challenging multidisciplinary cancer care in the different clinical settings of early, locally advanced, and metastatic disease, thus highlighting some priority areas of research.

## Graphical abstract

Cancer care is moving from a disease‐focused management toward a patient‐centered tailored approach. A huge technology‐driven revolution is taking place and innovative technologies are increasingly entering the mainstream of clinical practice to personalize cancer treatment. Here, we provide an outline of the role of modern radiation oncology in contemporary multidisciplinary treatment of cancer focusing on the main areas of innovation that are contributing to a shift toward an increasingly tailored use of radiotherapy.

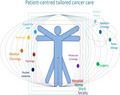

**Abbreviations**
4D‐RT: four‐dimensional radiotherapy
AI: artificial intelligence
CRT: conformal radiation therapy
IGRT: image‐guided radiotherapy
IMRT: intensity‐modulated radiation therapy
IORT: intraoperative radiotherapy
NCCN: National Comprehensive Cancer Network
NSCLC: non‐small cell lung cancer
SBRT: stereotactic body radiation therapy
VMAT: volumetric modulated arc radiotherapy

## 1 Introduction

Cancer is a global and primary health problem, with more than 18 million diagnosed cases and 9.6 million deaths worldwide each year (International Agency for Research on Cancer, 2019).

The value of multidisciplinary cancer to achieve best results for the patient care has been recognized since early nineties (Selby *et al*., 1996), and multidisciplinary cancer teams are now widely recommended as an essential instrument of effective cancer care policy by many leading scientific societies (Borras *et al*., 2015).

Together with surgery and systemic therapies, radiotherapy makes up a major component of cancer care. Based on evidence‐based radiotherapy indications, more than half of cancer patients during the course of their disease should receive at least one radiation treatment either alone or in combination with other treatment modalities (Borras *et al*., 2015). In early‐stage tumors, radiotherapy can be used alone for many tumor types as a radical, organ‐sparing treatment. In locally advanced cancers, radiotherapy can be used with curative intent either alone or combined with systemic therapies. Furthermore, radiotherapy can be administered either postoperatively, to increase the chance of local disease control, or preoperatively, to allow for less extensive surgery and better functional outcomes. In very advanced or metastatic cases, radiotherapy is very useful for easing cancer‐related symptoms.

Over the past two decades, cancer treatment has been revolutionized by two major changes. First, cancer care is moving from a disease‐focused management to a patient‐centered approach where all healthcare decisions and quality measurements are driven by the patient’s specific needs and desired health outcomes. Second, a dramatic technology‐driven revolution is taking place and innovative technologies are increasingly entering the mainstream of clinical practice to personalize cancer treatment. While these changes are expected to improve quality of cancer care, they are also remarkably increasing the complexity of decision making. Even if multidisciplinary teams and patient consent on an evidence‐based treatment plan would be desirable for every cancer patient, ‘real‐world’ provision of patient‐centered personalized cancer care might be constrained by practical issues due to limited knowledge and resources.

The aim of this review is to provide an outline of the role of modern radiation oncology in contemporary multidisciplinary treatment of cancer focusing on the main areas of innovation that are contributing to a shift toward an increasingly tailored use of radiotherapy. We will also highlight some practical issues which may challenge the provision of patient‐centered personalized radiation treatments in the different clinical settings of early, locally advanced, and metastatic solid tumors.

## 2 Radiation oncology and recent progress in cancer treatment

Over recent decades, mortality rates due to the major cancers, such as colorectal and breast cancers, have continued to decline in high‐income countries (Bertuccio *et al*., 2019), although the overall level and pace of improvement vary for each cancer type. Differences in the availability of, and access to, screening programs, as well as access to effective and high‐quality cancer care, are the likely causes of such differences (Arnold *et al*., 2019). There are several cancer types and stages for which survival rates have improved, including, for example, local‐stage esophageal cancer; regional‐stage female breast and colorectal cancer; and distant‐stage non‐Hodgkin lymphoma (Jemal *et al*., 2017). Improved surgical techniques and the centralization of surgical procedures in many countries have likely played a major role in the improved outcome for localized cancers (Latenstein *et al*., 2020; van Putten *et al*., 2018). Locally advanced cancers have mostly benefited from the increased use of combined multimodality approaches, including radiotherapy and chemotherapy (Berry *et al*., 2005; Brenner *et al*., 2012; Latenstein *et al*., 2020; Sant *et al*., 2006). For distant‐stage cancers, however, improvements in survival appear generally to be very small in absolute terms. One exception is metastatic non‐Hodgkin lymphoma, for which targeted therapies became available in the late 1990s (Schulz *et al*., 2007). For other distant‐stage metastatic cancers, patient prognosis remains poor, with treatment being provided exclusively for palliative reasons. Multidisciplinary management that aims to define individual, optimal treatment strategies is a fundamental aspect of high‐quality cancer care and often includes radiation oncology. Although the impact of multidisciplinary management on patient’s survival is still a matter of debate (Selby *et al*., 2019), there is clear evidence that a multidisciplinary approach to cancer care can increase the use of guideline‐based approaches and reduce time to treatment (Friedland *et al*., 2011).

During the last decade, technical advances in radiotherapy have made it possible to conform the high‐dose volume ever more accurately to the tumor shape in an ever easier, faster way and accessible way. Beyond technical evolution, the translation of biological knowledge into clinical treatment schedule is contributing to improved efficacy and safety of radiation therapy (Krause *et al*., 2020). More generally, when combined with other treatment modalities, radiotherapy can now cure a growing number of cancer patients and improve the chances of a patient’s long‐term survival, even for some previously incurable patients, such as inoperable patients with early stage non‐small cell cancers or oligometastatic patients (Jang *et al*., 2019; McClelland *et al*., 2017; Palma *et al*., 2019).

Beyond prolonging survival, radiotherapy can also improve the well‐being of patients by relieving symptoms or by preserving organs functions. More recently, the introduction of precision immunotherapy has completely changed the prognosis of patients with advanced disease, providing some with the chance of long‐term survival (Yu *et al*., 2019).

A list of most common indications for radiotherapy is provided in Table 2.

Despite the enormous progresses made in recent years in cancer care, there is still a pressing need to improve the quality and accessibility of care for patients with both early and advanced‐stage cancer. Although the practice of medicine is still largely empirical, we are at the dawn of precision oncology, where the choice of treatment is increasingly personalized as new predictive factors become available in clinical practice. In particular, three key areas are contributing to a shift toward an increasingly tailored use of radiotherapy in multidisciplinary cancer care: technology for radiation treatment planning and delivery; immunotherapy; and omics technologies, big data, and bioinformatics, which we discuss below.

## 3 Technology for radiation treatment planning and delivery

Technology has always had a central role in the continuous development of radiotherapy. As highlighted in specific papers of this thematic issue, nowadays a variety of technologies are in clinical use to treat patients (Fiorino *et al*., 2020; Grau *et al*., 2020). The ability to accurately delineate tumors continues to improve owing to the integration of existing and novel forms of computed tomography, magnetic resonance imaging, and positron emission tomography. Image guidance (Table 1) is increasingly entering the mainstream of radiation oncology practice. Technical advances are speeding up the process of tumor and healthy tissue contouring and treatment planning, thus making adaptive radiotherapy (Table 1) increasingly workable in routine clinical practice. These advances are progressively enabling the delivery of ever more effective radiation doses to tumors that are physically close to very radiosensitive, essential organs and structures. This is the case for inoperable pancreatic cancer where 4D image‐guided adapted stereotactic radiotherapy (Table 1) can support potentially curative surgery in some patients (Boldrini *et al*., 2019b; Chen‐Zhao *et al*., 2020; Rudra *et al*., 2019).

**Table 1:** A summary of key radiotherapy techniques.

Radiotherapy technique	Brief description
External beam radiotherapy	External beam radiotherapy is the most common form of radiotherapy. The patient lies on a couch, and an external source of ionizing radiation (either photons, electrons, or particles) is pointed at a particular part of the body
Brachytherapy	Brachytherapy is a form of radiotherapy where a sealed radiation source is placed inside, or next to, the area requiring treatment
Three‐dimensional conformal radiotherapy (3D‐CRT)	3D‐CRT is an advanced technique that incorporates the use of imaging technologies to generate three‐dimensional images of a patient's tumor and nearby organs and tissues to shape the radiation beams to match the shape of the tumor
Four‐dimensional radiotherapy (4D‐RT)	4D‐RT also called respiratory gating is a radiation treatment used to target tumors that move with a patient's breathing, such as lung, pancreatic, and other gastrointestinal cancers
Intensity‐modulated radiotherapy (IMRT)	IMRT is an advanced type of radiation therapy that enables precise conformation of the radiation dose to complex target shapes
Image‐guided radiotherapy (IGRT)	IGRT is the use of imaging during radiation therapy to improve the precision and accuracy of treatment delivery
Volumetric modulated arc radiotherapy (VMAT)	VMAT is a radiation therapy technique that delivers the radiation dose continuously as the treatment machine rotates. This technique accurately shapes the radiation dose to the tumor, while minimizing the dose to the organs surrounding the tumor
Proton therapy	Proton therapy is a type of external beam radiotherapy that uses a beam of protons
Stereotactic radiotherapy	Stereotactic radiotherapy is a method of external beam radiotherapy, in which a clearly defined target volume is treated with high precision and accuracy with a biologically high radiation dose (Guckenberger *et al*., 2020a)
Intraoperative radiotherapy (IORT)	IORT is a technique that involves precise delivery of a large dose of ionizing radiation to the tumor or tumor bed during surgery
Adaptive radiotherapy	Adaptive radiotherapy is defined as changing the radiation treatment plan delivered to a patient during a course of radiotherapy to account for temporal changes in anatomy
Spatially fractionated radiotherapy	Spatially fractionated radiotherapy is distinctive from the standard radiation approaches, as it treats the total tumor with a nonuniform dose, effectively treating the tumor while staying within normal tissue tolerance of the surrounding structures (Yan *et al*., 2019)
Flash radiotherapy	FLASH radiotherapy is distinctive from the standard radiation approaches as it involves the ultra‐fast delivery of radiation treatment at dose rates several orders of magnitude greater than those currently in routine clinical practice (Symonds and Jones, 2019)

While improved imaging and radiotherapy technology have allowed ablative doses to be delivered to most early‐stage cancers, the standard radiation dose schedule for many locally advanced tumors, such as for non‐small cell lung cancer (NSCLC), has been almost unchanged since the 1980s, when radiotherapy was delivered using a 2D technique (Perez *et al*., 1980). Indeed, most dose‐escalation attempts in inoperable NSCLC patients result in higher levels of acute and late toxicity, compared to the use of conventional chemoradiotherapy, even when using modern techniques (van Diessen *et al*., 2019). Whenever it is technically feasible, increasing the space between the tumor and the organs at risk is the most obvious solution and may lead to positive results. For example, this strategy has been successfully used to reduce rectal toxicity in prostate cancer patients, by placing an absorbable hydrogel spacer between the prostate and the rectum before irradiation (Karsh *et al*., 2018). Although intensity‐modulated radiotherapy (IMRT) (see Table 1 ) is the standard treatment, 3D‐conformal radiation therapy (CRT) (see Table 1) using a hydrogel spacer might be an alternative treatment option in these patients.

## 4 Immunotherapy

Immunotherapy has also revolutionized the treatment of some cancer patients, such as those with metastatic NSCLC, leading to unprecedented survival benefits in selected patients (Burtness *et al*., 2019; Herbst *et al*., 2018). Unfortunately, however, most patients do not benefit from immunotherapy owing to primary resistance. Accumulating evidence suggests that stereotactic radiotherapy might synergize with immunotherapy without increasing the toxicity, thus potentially overcoming immune resistance in some patients (Theelen *et al*., 2019). As highlighted in a specific paper of this thematic issue (Mondini *et al*., 2020), there is a growing number of preclinical and clinical data concerning the combination of radiotherapy with immunotherapy, in particular with immune checkpoint inhibitors. As an example, 98 patients with metastatic NSCLC, who had received photon radiotherapy prior to immunotherapy, showed significantly improved progression‐free survival and overall survival in a secondary analysis of a clinical landmark trial (Shaverdian *et al*., 2017).

However, little is still known about the effect of predictive factors, nor about the effect of radiotherapy dose, fractionation, timing, and treatment site on the antitumor immune response.

## 5 Omics technologies, big data, and bioinformatics

Digital health offers the chance to learn more than ever about what patients really want and how better to accomplish their will. Big data is an integral part of digital health transformation, which is profoundly changing the way medical data are generated and stored. Digital health can support continuously learning artificial intelligence (AI) platforms, which can integrate all available data (clinical, imaging, biological, genetic, cost) to produce validated predictive models and which can develop multifactorial decision support systems. In the future, continuously learning AI platforms might also enable the rapid integration of innovation into predictive models. The use of decision support systems based on big data is rapidly gaining importance in clinical practice, especially in complex fields of knowledge where numerous variables have to be considered at the same time, such as in a patient’s centered multidisciplinary approach to cancer care (McNutt *et al*., 2018).

The full personalization of oncological treatments (i.e., chemotherapy and radiotherapy) could not neglect the data generated by the different omics domains that will support clinicians in the choice of the most suitable therapeutic approaches, thereby reducing toxicities and overtreatments, and optimizing the use of all the available resources.

In addition to the growing awareness of how to use clinically large datasets of demographical, biological, and wet lab omics data, AI applications that use biomedical imaging data are gaining a dominant role in multiomics‐based clinical decision approaches (Boldrini *et al*., 2019a). In this context, radiomics is an innovative approach that enables high‐dimensional data to be extracted and interpreted from standard medical images (Lambin *et al*., 2012; Rizzo *et al*., 2018). Several studies in recent years have demonstrated the potential advantages of applying radiomics techniques to the clinical management of cancer; such studies have described the role of radiomics in characterizing the tumor through its quantitative analysis, describing its genetic signatures, disclosing its biological targets, and predicting the tumor’s response to multimodal treatments (Bodalal *et al*., 2018).

Unfortunately, however, radiomics variables are still burdened by numerous methodological and biological vulnerabilities that might hamper their effective integration into multidimensional, clinical decision support systems. Indeed, even for the same image, two different software implementations may produce different values. As such, standardization initiatives are needed to increase reproducibility of radiomics studies and facilitate clinical translation of radiomics (Zwanenburg *et al*., 2020).

Nevertheless, by taking full advantage of a multiomics approach to treating cancer, we can open new frontiers in cancer care, find innovative targets for therapies, and enhance patients’ quality of life. In addition to clinical trials, integration of real‐world multiomic data into continuously learning AI platforms may provide in the future a timely approach to generate evidence in rapidly evolving environments such as radiation oncology (Lievens *et al*., 2020).

State‐of‐art technologies, new drugs, and improved knowledge about treatment synergies may allow ever more precise treatments in the different clinical settings of early, locally advanced, and metastatic disease.

## 6 Radiation oncology in managing early‐stage cancers

The decision as to whether or not a patient should receive radiation therapy as part of their cancer management should be consistent with evidence‐based clinical practice guidelines (Borras *et al*., 2015). Owing to its noninvasive nature, radiotherapy represents a compelling alternative to surgery for many early‐stage tumors. Curative radiotherapy is usually the treatment of choice in cases where it can provide similar disease and survival outcomes as compared with surgery while preserving function (as in the case of early‐stage anal cancer that involves the sphincter) or where surgery has been ruled out due to a patient’s comorbidities or refusal (e.g., in inoperable early‐stage lung cancer). In cases where radiotherapy and surgery have similar outcomes in terms of overall survival but different risk/benefit profiles (as in early‐stage glottis and early‐stage prostate cancer Hamdy *et al*., 2016; Higgins *et al*., 2009), the best treatment option is a matter of debate in most cases.

While for many tumor sites radiotherapy alone can successfully cure most patients with early cancers (Hamdy *et al*., 2016; Higgins *et al*., 2009; Zheng *et al*., 2014), some of them may still experience local failure or high morbidity and complications because of different tumor and normal tissues radio‐sensitivity. Modern techniques allow to measure factors of radiation resistance or radiation sensitivity in patient tumors. The definition of patient groups based on biological risk factors, for which a very good or a very poor predicted outcome after standard treatment is expected, may support treatment decisions (Krause *et al*., 2020). As an example, although a quite infrequent but devastating one, early‐stage glottis cancer can recur locally after radical radiotherapy, requiring salvage laryngectomy in most cases, with a high risk of local re‐recurrence and complications. The determination of risk factors for radiation failure would be valuable and might provide better outcomes for those patients who might instead benefit from larynx surgery as a first option (Eskiizmir *et al*., 2016).

In a patient‐centered approach, the right choice between competing treatment options is that which best represents the optimal trade‐off between benefits and risks from the patient perspective. Therefore, patient’s empowerment is a key component of cancer care. Nevertheless, shared decision making can be a very complex process. Indeed, some patients may not wish to participate in choosing their own treatment (Leech *et al*., 2020). Moreover, information needs of cancer patients can be very heterogeneous and health professionals may not be completely aware on the topics of most importance for patients (Ruesch *et al*., 2014). As a consequence, some patients may perceive a lack of specific information and experience decisional conflict (Mokhles *et al*., 2017).

In order to offer proper counsel to patients, it is important to capture the relevant endpoints that matter most to them, in the context of each oncological setting and indication (Lievens *et al*., 2019). Traditional, trial‐based endpoints, such as survival, disease‐free survival, and safety, do not emphasize the patients’ perspective. This highlights the need for a new framework for shared decision making that focuses on patient‐centered endpoints. In the case of early‐stage glottis cancer, for example, the probability of retaining intelligible speech could be a key factor in a patient choosing between surgery and radiotherapy. Based on current knowledge, a patient could thus be counseled that three‐dimensional conformal radiotherapy (3D‐CRT, see Table 1) might retain better voice quality than would laser surgery (Dinapoli *et al*., 2010). However, for completeness of information, a patient should also be informed that this benefit might be limited to particular acoustic and perceptual outcomes (Lee *et al*., 2019) and that a personalized prediction of outcome cannot be given since improvements in voice quality scores are seen at the overall population, rather than on an individual level. In the future, digital health and AI may allow to counsel a patient with early‐stage glottis cancer by simply making him listen to what his voice will be like after radiotherapy or surgery.

Patients with early‐stage cancers who want an expeditious treatment may prefer surgery over radiotherapy (Stoeckli *et al*., 2003). To improve patient convenience, the number of treatments sessions and/or the length of treatment can be reduced by choosing hypofractionated schedules. This approach can also lower treatment costs (Moore *et al*., 2020). As an example, according to international guidelines (NCCN, 2020) four different dose‐fractionation schedules may be used as appropriate for early‐stage T1N0 glottis cancer, with different treatment durations ranging from 3 to more than 6 weeks. Among hypofractionated radiation treatments, stereotactic body radiation therapy (SBRT, see Table 1) seems to be effective and safe for treating laryngeal cancer with only five treatment fractions (NCT01984502, ClinicalTrials.gov). While all these regimens have similar overall outcomes in different groups of patients with early‐stage glottis cancer, some tumor or patient’s specific factor may affect the individual risk/benefit profile of different dose‐fractionation schedules. Translation of biological research into clinical practice may allow in the future individualization of radiation doses or fractionation schedules (Krause *et al*., 2020).

Treatment selection based on predictive biomarkers and focused on patients needs would be highly desirable to deliver excellent cancer care. Modern radiation treatments can be increasingly customized to meet the needs of many patients with early‐stage solid tumors. Contemporary radiotherapy is a noninvasive and ever more effective, safe, and expeditious option of cure for many early‐stage tumors (some examples are provided in Table 2).

**Table 2:** Cancers commonly treated with radiotherapy (NCCN, 2020; Palma *et al*., 2019).

Tumor site	Disease stage	Treatment intent	Radiotherapy technique	Other therapies used in combination
Head and neck	Early and locally advanced	Radical curative	IMRT or other conformal techniques (3D‐CRT, helical tomotherapy, volumetric modulated arc therapy, proton beam therapy) depending to stage, tumor location, physician training experience, and available physics support	Chemotherapy and/or primary surgery in locally advanced disease
Prostate	Early and locally advanced	Radical curative	Highly conformal RT techniques such as IMRT, proton beam therapy, or brachytherapy (in low‐risk disease)	Hormonal therapy in locally advanced disease
Bladder	Early and locally advanced	Radical curative	Conformal radiotherapy with daily image guidance	Endoscopic resection in muscle invasive prior to radiotherapy whenever feasible. +/− concurrent chemotherapy
Cervix	Early and locally advanced	Radical curative	Conformal radiotherapy with daily image guidance and intracavitary or interstitial brachytherapy	Concurrent chemotherapy in locally advanced disease
Breast	Early and locally advanced	Radical curative	Conformal external beam radiotherapy (electrons or brachytherapy may also be used for the boost volume)	Surgery +/− adjuvant or neoadjuvant chemotherapy +/− adjuvant hormonal therapy
Lung	Limited‐stage small cell lung cancer	Radical curative	3D‐CRT as minimum technological standard. IMRT, VMAT, IGRT, motion management, and proton therapy are appropriate	Chemotherapy
	Early and locally advanced NSCLC	Radical curative	3D‐CRT as minimum technological standard. IMRT, VMAT, IGRT, motion management, and proton therapy are appropriate	Chemotherapy and immunotherapy in locally advanced disease
Esophagus	Early and locally advanced	Radical curative	3D‐CRT as minimum technological standard. IMRT and proton therapy are appropriate	Concurrent chemotherapy, +/− surgery
Rectum	Locally advanced	Radical curative	3D‐CRT IMRT and SBRT in the setting of a clinical trial of re‐irradiation IORT in some cases	Surgery; +/− concurrent chemotherapy
Anal canal	Early and locally advanced	Radical curative	IMRT with daily image guidance	Concurrent chemotherapy
Any	Locally advanced and metastatic (symptomatic patients with poor life expectancy and/or large tumor burden)	Palliative	External beam radiotherapy	Supportive care; +/− Systemic therapies
	Oligometastatic	Improvement of survival	Stereotactic radiotherapy	–

## 7 Radiation oncology in managing locally advanced cancers

The standard approach for treating locally advanced malignancies usually consists of a combination of radiotherapy and systemic therapies. Surgery is also an essential part of an integrated approach in some cases, such as when treating breast, esophageal, and rectal cancer.

As with the treatment of early‐stage tumors, the treatment of locally advanced cancer tends to be increasingly tailored to the individual patient to minimize the risk of local recurrence and distant metastases through multidisciplinary collaboration. Moreover, patient preferences and quality of life preservation are increasingly emphasized in the decision‐making process and in treatment strategy designs.

As an example, at the beginning of the last century, William J. Mayo did not advocate sphincter‐saving procedures for the treatment of rectal cancer because this strategy would not be radical enough, although he acknowledged that some rectal cancer patients might choose to maintain fecal continence even at the price of a possible decreased survival (Mayo, 1916). In the present day, neoadjuvant chemoradiotherapy allows for sphincter‐saving surgery in three quarters of the patients. Moreover, in almost one third of the patients the tumor completely disappears after neoadjuvant chemoradiotherapy. These patients who exhibited a complete response as a rule prefer a non‐operative, watch‐and‐wait approach. Accordingly, increasing numbers of patients, who respond very well after neoadjuvant chemoradiotherapy, are managed non‐operatively (Gosselink, 2019).

Progress in radiotherapy technology has dramatically improved our ability to customize the radiation dose to each particular anatomic situation, thus enabling tumors to be exposed to escalating doses of radiotherapy while sparing the surrounding normal tissue. Nevertheless, although increasing doses can theoretically improve patient outcomes, they might not be feasible for some patients, even with modern irradiation techniques, because the radiation dose needed to eradicate a tumor exceeds that tolerated by the organ at risk. A winning strategy to lower radiation doses is to exploit the synergy between radiotherapy and other treatment modalities. In the last two decades, the greatest improvement in the survival of patients with inoperable, locally advanced, NSCLC has come from immunotherapy. In a recent randomized trial, maintenance immunotherapy after conventional radio‐chemotherapy with a standard dose resulted in significantly longer disease‐free and overall survival rates, relative to treatment with a placebo (Antonia *et al*., 2018).

New unconventional treatment modalities, such as spatially fractionated radiation therapy and flash radiotherapy (see Table 1), might further widen the therapeutic window by increasing the biologically effective radiotherapy dose to a tumor (Bai *et al*., 2018), and synergizing with immunotherapy (Billena and Khan, 2019).

Modern radiation therapy technology and the synergy with new drugs may increase the effectiveness of integrated treatments for locally advanced cancer and open the frontiers for a role of radiotherapy beyond palliation also in metastatic patients.

## 8 Radiation oncology in managing metastatic cancers

Until recently, radiation therapy had primarily been used in the context of the multidisciplinary management of patients with metastatic disease to palliate and ease symptoms. However, it is becoming increasingly evident that ablative metastasis‐directed therapies, when included as a standard of care, can provide long‐term survival in some metastatic patients. In particular, metastasis‐directed stereotactic radiotherapy can prolong survival in some ‘oligometastatic’ patients, who have a maximum of three to five metastases on imaging (Gomez *et al*., 2019; Iyengar *et al*., 2018; Ost *et al*., 2018; Palma *et al*., 2019; Ruers *et al*., 2017). Unfortunately, neither ‘oligometastatic’ disease‐specific biomarkers nor prospectively validated prognostic scoring systems yet exist (Pitroda and Weichselbaum, 2019) so it remains impossible to identify patients with truly limited metastatic capacity, who might really benefit from such a radical approach. Furthermore, the current definition of ‘oligometastatic’ status is based solely on the number of metastases on imaging. It is therefore a broad status that might consist of patients with very different prognoses and treatment options. For example, a patient with ‘*de novo*’ oligometastatic NSCLC, and a patient with an initial, polymetastatic NSCLC and residual oligometastatic disease after first‐line systemic therapy, both fall under the same definition of ‘oligometastatic’, although being very different from a clinical perspective. To account for the different timepoints in the history of oligometastatic patients, a recent classification of the oligometastatic status has been proposed by an international consensus of 20 experts (Guckenberger *et al*., 2020b). The prognostic value of this classification will be assessed in the ongoing OligoCare prospective cohort trial (NCT03818503, ClinicalTrials.gov).

Following the introduction of immunotherapy, the role of radiotherapy is evolving beyond that of palliative care in patients with widespread metastatic diffusion as well. Indeed, owing to its immune‐modulatory effects, stereotactic radiotherapy might also be used to overcome refractoriness to immunotherapy in some patients (Ho *et al*., 2020; Maity *et al*., 2018).

Technological developments in radiotherapy are continuing apace and are likely to confer further clinical benefit, even in metastatic patients, particularly in combination with immunomodulatory drugs.

## 9 Conclusions

The main aim of a physician dealing with a cancer patient is to provide him or her with the most appropriate individual treatment solution. While this patient‐centered approach is expected to improve quality of cancer care, the complexity of decision making is also remarkably increasing. Nowadays, radiotherapy can meet the needs of many cancer patients by providing a noninvasive, effective, safe, and expeditious cure. In patients with early‐stage cancer, due to its no invasive nature and increasing efficacy, radiotherapy is becoming an ever more effective treatment modality. Combined with surgery and systemic therapies, such as immunotherapy, radiotherapy can now cure a growing proportion of patients with locally advanced tumors, and it can play a role beyond palliation also in patients with metastatic cancer. Multidisciplinary teams should make any effort to address patient’s needs by tailoring treatment choices based on predictive biomarkers, if available. Large prospective datasets may increasingly help multidisciplinary teams making individualized recommendations based on ‘real‐world’ results.

## Conflict of interest

The authors declare no conflict of interest.

## Author contributions

All authors contributed substantially to the conception, drafting, and revising of the work. VV and MM conceived the outline of the study. LB and SM reviewed the literature and drafted the manuscript. All authors reviewed and approved the final manuscript.
